# Exploring effective implementation pathways to become an excellent chief financial officer in public hospital: a qualitative comparative analysis (QCA) from China

**DOI:** 10.1186/s12913-024-10588-x

**Published:** 2024-01-23

**Authors:** Hongzhi Wang, Junjun Li, Xin Xiang

**Affiliations:** 1 Research Center of Hospital Management and Medical Prevention, Guangxi Academy of Medical Sciences, The People’s Hospital of Guangxi Zhuang Autonomous Region), Nanning, China; 2 The People’s Hospital of Guangxi Zhuang Autonomous Region, Nanning, China; 3 Institute of Fiscal and Finance, Shandong Academy of Social Sciences, 56 Shungeng Road, 250002 Jinan, Shandong China

**Keywords:** Hospital CFO, Competency, Qualitative comparative analysis, Effective implementation pathway, Hospital management

## Abstract

**Background:**

Hospital chief financial officer (CFO) contributes to improving health system performance. However, how to become an excellent hospital CFO has rarely been considered from a holistic perspective. This paper aims to identify competencies required by hospital CFO to fulfil the position’s responsibilities and explore effective implementation pathways to generate high performance and improve healthcare service.

**Methods:**

We conducted 61 semi-structured interviews with individuals in key leadership positions in China’s hospitals and researchers focusing on healthcare system management to identify core competencies necessary for hospital CFO. Interviews were analysed through a multi-stage review process and modified via expert vetting using a national panel of 23 professors. Subsequently, interviews were conducted with 32 hospital CFOs from 14 provinces throughout September 2021 to May 2022. We scored the performance of 32 hospital CFOs in various aspects of competency and used the fuzzy-set qualitative comparative analysis to explore the competency configurations of excellent CFOs.

**Results:**

We identify seven core competencies necessary for a hospital CFO to fulfil management practices, including personal morality, resource management, strategy management, learning ability, negotiating skill, leadership skill, and financial management. The findings indicate that a single competency factor is not a necessary condition to become an excellent hospital CFO. The results of qualitative comparative analysis then make it possible to propose four configurational paths, namely, supportive, interpersonal, all-around development, and technical, to become an excellent hospital CFO and achieve effective managerial performance.

**Conclusions:**

The responsibilities of hospital CFOs are complex and varied, hence, a better understanding of competencies required by CFO is essential to implement their responsibilities effectively. The identification in this study of the four effective implementation pathways to becoming an excellent hospital CFO enriches the literature on hospital management and provides implications for China’s hospitals and their CFOs.

**Supplementary Information:**

The online version contains supplementary material available at 10.1186/s12913-024-10588-x.

## Background

The chief financial officer (CFO), as a core member of the hospital management team, plays the primary role in improving health system performance, efficiently allocating hospital resources, sustainable strategic development, economic decision-making and leadership [1–3]. The COVID-19 crisis, with huge pressure on public finance, has particularly highlighted the responsibility of the CFO to provide an economically sustainable strategic focus to support healthcare improvements [2]. The advanced healthcare system, for example, the National Health Service (NHS) in the United Kingdom, has long placed the position of CFO in hospital to exercise the managerial functions of corporate governance and financial leadership. In contrast, an unadvanced healthcare system, likely China, has recently begun to highlight the development of the CFO position. Although China has published documents to supervise tertiary public hospitals in setting up CFO positions to meet the needs of hospital change management [4], the report published by the National Health Commission of China in December 2022 revealed that only 76% of tertiary public hospitals have set up the CFO position [5]. Even in this case, many hospitals still need to work on challenge that the appointed CFO could not be competent to fulfil the responsibilities and requirements of the position [2].

Hospital CFOs in China are primarily responsible for organizing the hospital to implementing relevant national laws and regulations, abiding by financial discipline, strengthening financial management and supervision, and protecting state property, as well as playing a supervisory and decision-making supporting role in significant matters such as operation management, business development, capital construction and capital operation of the hospital [1, 6]. Similar to CFOs in the NHS required qualifications and experience, hospital CFOs in China must be qualified accountants who are members of either the senior accountant or the certified public accountant classification and are suitably qualified and have relevant experience [7]. The aim of setting those essential attributes is to identify the suitable person with competencies in knowledge, skill, ability, and other characteristics necessary for an individual to meet the responsibilities of the CFO position. However, many hospitals and their CFOs need to understand the responsibilities of the CFO position fully and even equate them with the financial director [2]. It is no exaggeration to say that most hospital CFOs in China must have necessary competency to meet the position’s needs.

Hospital management is a complex system and refers to multiple perspectives [8]; hospital CFOs are required to respond to different situations using a variety of competencies to ensure the continuing delivery of financial duties and other objectives [2, 9]. A competency framework systematically defines the specific competencies required by the position, helps the organization evaluate, select, and train excellent candidates, and guides individual self-improvement [10, 11]. Competence-oriented development, in the context of the healthcare system, has been applied to various fields, such as medical leadership [12], academic physician [13], clinician [14], and nursing [15]. Despite the significant role played by hospital CFOs has been mentioned and several studies conducted to explore the competency framework of hospital CFOs [2, 16], the simple competency framework is difficult to deeply understand in hospitals, so the driving path to improve CFO performance needs to be clarified. Meanwhile, there is common no single optimal path to becoming an excellent individual in specific position; it may involve different combinations of competency factors and other complex mechanisms to encourage the individual to achieve high performance [17]. Hitherto, studies have yet to provide empirical evidence to indicate how various competencies affect hospital CFOs to produce high performance and identify effective implementation pathways to become excellent hospital CFOs in China.

Therefore, we aimed to fill this gap by addressing the following questions: What competencies should hospital CFOs possess in China? What competency configurations promote an individual to become an excellent hospital CFO? What are effective implementation pathways to improve the development of hospital CFO?

To address research questions, we introduce the method of qualitative comparative analysis (QCA), which is an approach developed by Charles Ragin [18, 19] to bridge the gap between quantitative and qualitative methodologies and is suitable for studies involving causal complexity [20–22]. The QCA method assumes that antecedents influence each other, rather than independently and focuses on the combinatorial effects of antecedent conditions by identifying configurations of necessary and sufficient conditions [23–26]. It has the advantage of exploring the possibilities of multiple configurations resulting in the same outcome and revealing causal asymmetry to the success or failure of outcomes [24, 25, 27]. As the effective method to address causality in complex systems, QCA is a relatively new approach for public health research [28]. To the best of our knowledge, this study pioneered the QCA method into human resource studies in health field.

## Methods

QCA can be used in descriptive and explanatory research, such as summarizing data, creating typologies, checking the coherence of subset relations, assessing hypotheses, testing atheoretical conjectures, and creating new theories [29]. As a holistic approach, QCA is a case-based perspective that considers each case complex. To explore the complexity in social sciences, QCA supports the concept of multiple conjunctural causation, specifically, (1) it commonly exists a combination of conditions (independent variables) generated the outcome or phenomenon (dependent variable); (2) the same outcome can be developed by several different combinations of conditions; (3) the given condition may have different effects on the outcome varying with contexts [27]. Hence, QCA provides different causal paths which lead to the same outcome. There are three types of QCA, including fuzzy-set QCA (fs/QCA), crispy-set QCA (cs/QCA) and multi-valued QCA (mv/QCA). To analyze the effective pathway implementation to improve the performance of hospital CFOs, we apply an fs/QCA in this study. Compared with cs/QCA and mv/QCA, an fs/QCA conditions identification uses membership degree assignment, focusing more on case-oriented and enables a more detailed explanation of the causal factors, which improves the research quality [30]. The steps of fs/QCA are identifying and calibrating causal variables, analyzing the necessity and sufficiency of conditions and discussing the results [31].

### Conditions identification

Hitherto, the competency of hospital CFO in China remains an under-explored topic in the literature; hence, to identify the conditions as competency necessary for hospital CFOs to fulfil position responsibilities effectively, semi-structured interviews were conducted with individuals in key leadership positions in China’s hospitals and researchers who focus on healthcare system management. Of the 89 invited, 61 respondents (68.54%), including 32 CEOs and CFOs from 21 tertiary hospitals and 29 researchers from 13 medical universities across 13 Chinese provinces, participated in this study. Most respondents (38; 62.30%) were males, and PhD degree holders were larger in number, with 39 respondents (63.93%). Table 1 presents the demographic characteristics of interviewees in this phase.

**Table 1:** Demographic characteristics of interviewees

Demographic characteristics	Number	Percent
**Gender**		
Male	38	62.30%
Female	23	37.70%
**Educational Status**		
Bachelor	5	8.20%
Master	17	27.87%
Ph.D	39	63.93%
**Profession type**		
Researcher	29	47.54%
Hospital CEO	17	27.87%
Hospital CFO	15	24.59%
**Age**		
30–40	15	24.59%
41–50	22	36.07%
51–60	18	29.51%
Above 60	6	9.83%

The interviews were conducted between January and July 2021 via face-to-face or video call. Participants were asked to read a summary of the scoping review before their interviews. After a short introduction to the concept of competency, participants were first asked to describe their understanding of hospital CFO’s position. Then, they were asked to describe the excellent hospital CFOs in their opinions. Furthermore, they also needed to provide the competency necessary for hospital CFOs to practice hospital management and explain those items. The interview guides in this stage was provided in Appendix 1.

Each interview was approximately 45 min, and with respondents’ permission, it was recorded. We transcribed, anonymized, transcribed and imported the interviews into NVivo. All interview transcripts were analyzed, combining deductive and inductive coding. Two researchers coded the original interview data, identified the initial concepts related to the competency of hospital CFOs, and then compared the results with those of a third researcher. After coding and analyzing, eight competencies were generated: personal morality, financial management, risk management, strategy management, leadership skill, learning ability, negotiating skill and resource management.

To further assess the applicability of categories of coding results, we applied expert vetting to reach a consensus. An expert panel comprises 23 professors in hospital management processing three rounds. In round 1, the initial competencies was sent to the panel, requiring modification of those items. We provided two open-ended questions: (1) Please provide comments or modifications for the proposed competencies of the hospital CFO; (2) Please provide additional suggestions for competencies necessary for the hospital CFO. According to results derived from round 1, a questionnaire with the five-point Likert scale (1 = strongly disagree; 2 = disagree; 3 = undecided; 4 = agree; 5 = strongly agree) was developed and used in round 2 and 3 to obtain the extent to which they agreed or disagreed with those competencies. According to the meaningful comments from round 1, we adjusted risk management ability integrated into strategy management ability. After rounds 2 and 3, seven competencies were identified, including personal morality, resource management, strategy management, learning ability, negotiating skill, leadership skill and financial management. Table 2 reports the competencies required by the hospital CFO and the statistics for each item in round 2 (in brackets) and round 3. Additionally, the results of testing the statistical significance for the differences in the mean scores in the two rounds are also presented in Table 2.

**Table 2:** Competency for CFO from the Delphi study

Competency	Mean
Personal Morality (PM)	4.43^***^
	[4.39]
Resource Management (RM)	4.61^***^
	[4.48]
Strategy Management (SM)	4.35^***^
	[4.35]
Learning Ability (LA)	4.61^***^
	[4.48]
Negotiating Skill (NS)	4.26^***^
	[4.17]
Leadership Skill (LS)	4.44^***^
	[4.30]
Financial Management (FM)	4.35^***^
	[4.35]

Notes: It reports the mean values in Round 3 first and those in Round 2 in brackets; a t test was used to test whether the means of competencies of Round 3 have statistical significance compared with Round 2. *** denotes that the mean value is different between rounds with statistical significance levels of 1%

### Data collection and sample

The sample in this study was an individual who served in a CFO position in a tertiary hospital. The following principles guided our recruitment strategy: (1) an individual had over two years of working experience in the hospital CFO position; (2) the geographical distribution of the candidates covers different provinces; (3) we could from a contract with them. We invited 46 potential interviewees, of which 69.60% (32 individuals) agreed to participate. Most participants had master’s degrees (90.63%) and were men (87.50%). Additionally, scholars have indicated the positive association between economic development level and concentration level of medical resources [32]; considering the different concentration levels of medical resources and their effect on hospital management, we invite respondents from different regions. Participants came from 14 provinces with high concentration level of medical resources (e.g., Beijing, Shanghai and Guangdong), where GDP per capita ranked among the top ten in China, medium concentration level of medical resources (e.g., Anhui, Shanxi and Hebei) where GDP per capita ranked 11–20 and low concentration level of medical resources (e.g., Guizhou, Guangxi and Yunan) where GDP per capita ranked below 20. The interviews were conducted throughout September 2021 to May 2022 (Table 3).

**Table 3:** Study samples for QCA

Interviewee	Age	Gender	Education	Region	Duration	Date of interview
1	46–50	Male	Master	Beijing	1 h 20 min	Sep-21
2	41–45	Female	Master	Beijing	59 min	Nov-21
3	41–45	Male	Master	Beijing	1 h 7 min	Jan-22
4	46–50	Male	Master	Beijing	1 h 13 min	Feb-22
5	51–55	Male	Master	Shanghai	56 min	Oct-21
6	46–50	Male	Master	Shanghai	1 h 11 min	Oct-21
7	51–55	Female	Master	Shanghai	1 h 13 min	Feb-22
8	51–55	Male	Bachelor	Guangdong	1 h 30 min	Nov-21
9	46–50	Male	Master	Guangdong	1 h 15 min	Apr-22
10	46–50	Male	Master	Jiangsu	55 min	Nov-21
11	46–50	Male	Master	Jiangsu	52 min	Dec-21
12	51–55	Male	Bachelor	Shandong	1 h 21 min	Sep-21
13	46–50	Female	Master	Shandong	1 h 03 min	Nov-21
14	46–50	Male	Master	Shandong	1 h 16 min	May-22
15	51–55	Male	Master	Anhui	59 min	Oct-21
16	51–55	Male	Bachelor	Anhui	1 h 27 min	Mar-22
17	46–50	Male	Master	Hubei	1 h 11 min	Nov-21
18	41–45	Female	Master	Hebei	50 min	Sep-21
19	51–55	Male	Master	Hebei	1 h 10 min	Nov-21
20	41–45	Male	Master	Jiangxi	1 h 17 min	Sep-21
21	46–50	Male	Master	Jiangxi	1 h 21 min	Jan-22
22	51–55	Male	Master	Guizhou	52 min	Mar-22
23	46–50	Male	Master	Shanxi	1 h 02 min	Sep-21
24	46–50	Male	Master	Shanxi	1 h 22 min	Dec-21
25	41–45	Male	Master	Sichuan	58 min	Oct-21
26	51–55	Male	Master	Guangxi	50 min	Jan-22
27	41–45	Male	Master	Guangxi	1 h 03 min	Jan-22
28	46–50	Male	Master	Guangxi	1 h 29 min	Feb-22
29	46–50	Male	Master	Guangxi	55 min	Feb-22
30	46–50	Male	Master	Hunan	1 h 11 min	Sep-21
31	46–50	Male	Master	Yunnan	1 h 09 min	Oct-21
32	51–55	Male	Master	Yunnan	1 h 20 min	May-22

The interviews were conducted face-to-face whenever possible. Due to the very tight schedule of the participants and the geographical distance, part of the interview was conducted via video conference. The interview grid included three main themes: (1) presentation of interviewee and his/her background; (2) introducing an understanding of the CFO position and sharing the work experience in this position; (3) the understanding for seven core competencies and how they implement those competencies in their work. The interview guides in this stage are provided in Appendix 2. The interviews were recorded, after a formal consent from was obtained from the respondents. The 2187 min of interviews were fully transcribed and anonymized.

Two researchers used NVivo11 software to conduct simultaneous sentence-by-sentence analysis and coding of the original interview data to identify specific items and performance of each respondent on the core competencies. After initial coding, the coding results completed by two researchers were compared to ensure reliability and validity. If there was a dispute over the coding result, the third researcher coded it and then selected similar coding as the final result.

### Calibration of causal variables

#### Competency index score

First, based on the interview data, researchers rated the participants on their mastery of those core competencies. The score of hospital CFOs score who had thoroughly mastered these competencies was 5, and the score of those who had not mastered them was 1. The score varied between 1(Completely absent) and 5(fully mastered). Since each competency item could be mentioned several time in an interview, the average score of each competency of each interviewee was calculated:

$$x = \frac{{(1 \times {\text{a}} + 2 \times {\text{b}} + 3 \times {\text{c}} + 4 \times {\text{d}} + 5 \times {\text{e}})}}{{{\text{a}} + {\text{b}} + {\text{c}} + {\text{d}} + {\text{e}}}}$$

where a is the completely absent frequency, b is the absent frequency, c is neither absent or mastered frequency, d is the mastered frequency, and e is the fully mastered frequency.

#### Outcome classification

As the development of hospital CFO is just emerging in China, there is no mature assessment system to evaluate the post performance of CFO. Hence, in this study, researchers rated the participants’ performance in their positions based on the interviews, using a scale from 1 to 5, with higher values representing better performance. After the initial rating of performance, we also invited ten experts to verify each participant’s rating and reach a consensus.

#### Calibration

Calibration is described as the process of transforming variables into set membership, referring to (1) the threshold for full set membership that is calibrated as 1; (2) the threshold for full set non-membership that is calibrated as 0; and (3) the crossover point indicating maximum ambiguity that is calibrated as 0.5 [33–35]. Following existing studies [36], we adopted the principles of direct calibration [20] to calibrate the variables. Specifically, we calibrated the values above the 95th percentile for the ‘fully in’, the values below the 5th percentile for the ‘fully out’, and the median or 50th percentile for the ‘crossover point’. Table 4 reports the calibrations information of each condition item and outcome item. For example, based on the all interview data, the item of personal morality was 5 for the 95th percentile, 3 for the 50th percentile and 2 for the 5th percentile. Hence, its value for fully in, crossover point and fully out was 5, 3, and 2, respectively. After confirming the calibrations of each condition and outcome variable, we used the fsQCA 3.0 software to compute the variable for each interviewer.

**Table 4:** Calibration of conditions and outcome

Outcome/Conditions	Calibration		
	Fully in	Crossover point	Fully out
Personal Morality	5	3	2
Resource Management	5	3	1
Strategy Management	5	2	1
Learning Ability	5	3	1
Negotiating Skill	5	3	1
Leadership Skill	5	3	1
Financial Management	5	4	1
Performance	5	4	1.55

## Results

### Necessity conditions analysis

The fsQCA 3.0 software was performed to detect the necessity and sufficiency conditions. The necessity of a single condition should be tested before exploring the sufficient configurations of conditions for the outcome [20]. Typically, the consistent threshold of necessary conditions was set as 0.9 [30, 35]. The results (Table 5) indicate that the consistency of the seven antecedent conditions and their non-conditions did not reach the critical value of 0.90. Hence, there was no necessary condition in this study. It indicates that CFO performance cannot be determined by one factor, and multiple factors should be taken into consideration.

**Table 5:** Necessity test of single conditions

Causal Conditions	High Performance		General Performance	
	Consistency	Coverage	Consistency	Coverage
PM	0.649	0.479	0.772	0.725
~ PM	0.627	0.684	0.445	0.617
RM	0.825	0.675	0.495	0.515
~ RM	0.407	0.388	0.687	0.833
SM	0.789	0.723	0.391	0.455
~ SM	0.406	0.344	0.763	0.821
LA	0.835	0.635	0.567	0.548
~ LA	0.406	0.425	0.623	0.827
NS	0.734	0.610	0.521	0.550
~ NS	0.458	0.429	0.630	0.751
LS	0.814	0.686	0.484	0.519
~ LS	0.429	0.396	0.707	0.829
FM	0.666	0.688	0.493	0.647
~ FM	0.658	0.505	0.762	0.744

Note: (~) denotes the absence of the condition

### Analysis of sufficiency

**Fig. 1:**
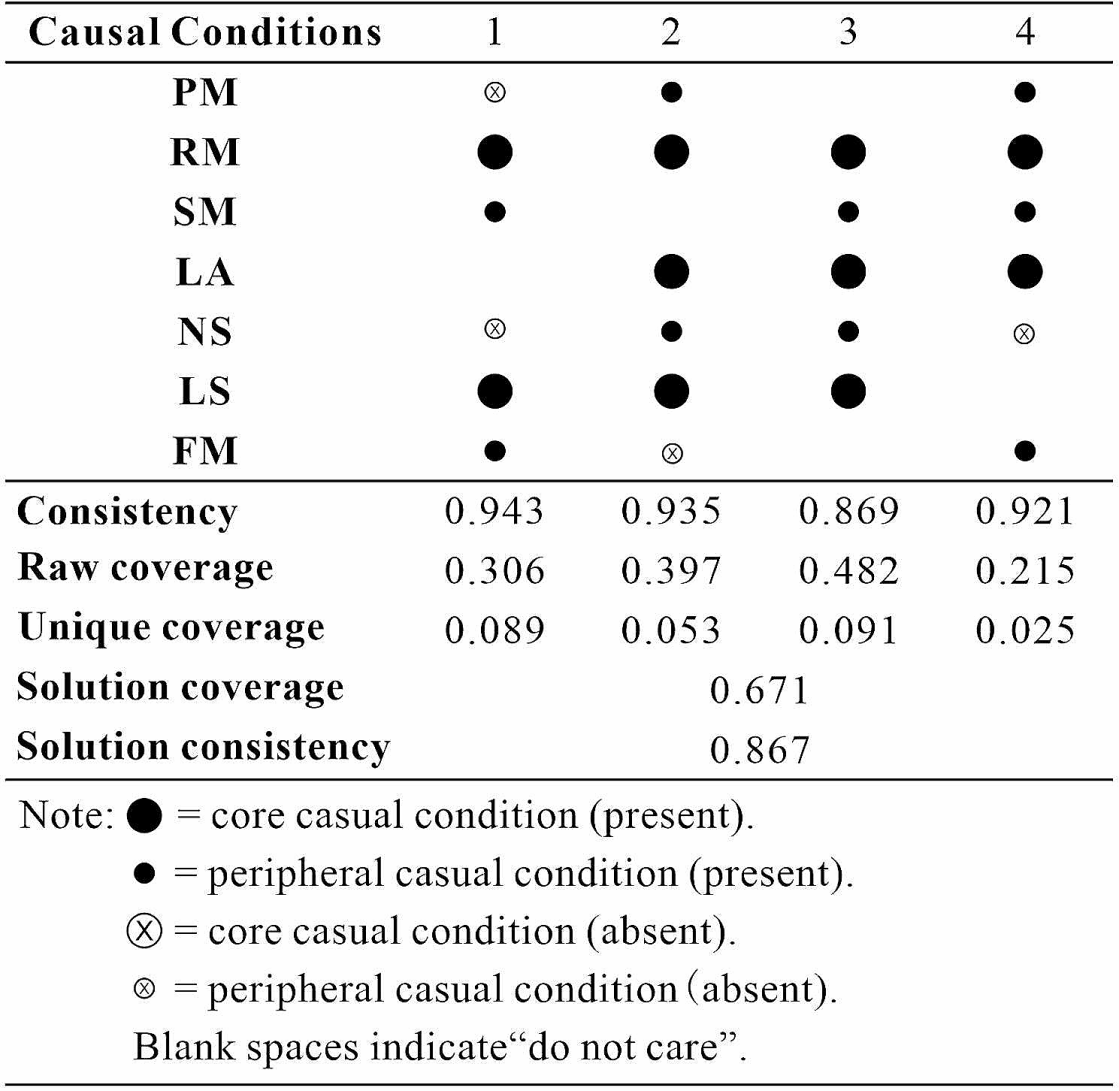
Analysis of sufficient conditions

The sufficiency analysis of conditional configurations is to determine whether there is a combination of conditions that is sufficient for an outcome to occur [37, 38]. Figure 1 shows the results of the configuration analysis of seven competency conditions on the performance of the hospital CFO. The coverage of the overall solution is 0.671, indicating that a greater number of empirical cases are covered. The overall consistency is 0.867, and the consistency scores of the four configurations are all grater than 0.80, indicating the significance level of all configurations.

In configuration 1, the presence of resource management and leadership skill is the core condition, strategy management and financial management exist as peripheral conditions. Despite the absence of personal morality and negotiating skill as a peripheral condition, an individual can become an excellent hospital CFO. This path is called the “supportive type” because those conditions better reflects the changed-oriented of effective leadership, which emphasizes that leaders articulate an inspiring vision and lead others to achieve organization vision [39]. In configuration 2, three conditions are core conditions including resource management, learning ability, and leadership skill. In contrast, personal morality and negotiating skill are peripheral conditions, and only financial management does not exist as a peripheral condition. We called this path “interpersonal type” because it reflects the relation-oriented of effective leadership, emphasizing that leaders build good cooperative relationship with others [40]. In configuration 3, three core conditions exit in this path, including resource management, learning ability and leadership skill, and two peripheral conditions, including strategy management and negotiating skill. We called this path “all-around development type” because it includes most competencies and reflects flexible leadership, emphasizing that leaders can create value by balancing multiple choices [41]. In configuration 4, resource management and learning ability exist as core conditions, and personal morality, strategy management and financial management exist as peripheral conditions. Negotiating skill is absence as peripheral condition in this path. This path is called the “technical type” because it better reflects task-oriented of effective leadership, emphasizing that leaders can ensure that people and other resources are used efficiently to achieve job objectives [42].

### Robustness test

To ensure the reliability of the results, the common methods to assess the robustness test include changing the consistency threshold level, adding or removing samples, and adding other conditions [43]. Concerning the above methods and following the existing studies [30], we first adjusted the consistency threshold level from 0.80 to 0.75 and found that the four types were still supported. There was a slight decrease in overall consistency and a slight increase in overall coverage. Second, three sample cases were randomly selected and eliminated. The solutions remained similar, indicating that the findings remain robust.

## Discussion

Based on semi-structured interview, our study identified seven core competencies necessary for hospital CFOs to practice position responsibilities, including personal morality, resource management, strategy management, learning ability, negotiating skill, leadership skill and financial management. Subsequently, fsQCA was conducted, and a truth table algorithm was used to identify sufficient solutions. The results indicate that the excellent hospital CFO cannot be achieved through a single factor but can through multiple and equivalent configurations of these causal condition variables.

### Competency index

This study identifies seven competencies necessary for hospital CFOs to practice their responsibilities. Specifically, personal morality (PM) refers to the principles and values to behaving ethically because CFO needs to do the right things and play a moral role in leading hospital finance staff. Resource management (RM) describes the CFO’s ability to reasonably arrange and allocate resources to appropriate projects and departments to improve resource use effectively, highlighting responsibility in stewardship of public finance and resource. Strategy management (SM) refers to the CFO’s ability to anticipate and seize an opportunity and change, emphasizing that the CFO plays a core role in developing organization’s vision and strategic direction. Learning ability (LA) describes the CFO’s awareness of self-improvement and constant learning of new techniques and knowledge, ensuring financial expertise and advice at both strategic and operational levels. Negotiating skill (NS) refers to the ability to interact with external stakeholders on behalf of the hospital, ensuring negotiations with other organizations efficiently. Leadership skill (LS) describes the ability to develop good relationships with partners, internal members, and external stakeholders and to motivate people to provide high-quality healthcare. Financial management (FM) refers to the ability and knowledge to develop and maintain an effective and comprehensive system of internal financial control, consistent with the primary functional role of the CFO in formulating, implementing and monitoring financial strategies.

### Competency configuration analysis

The hospital operates in a complex system and requires excellent CFOs to play significant corporate leadership and management roles to improve healthcare service. Based on the QCA results, this study obtained four configurations to being excellent hospital CFOs, namely “supportive type”, “interpersonal type”, “all-around development type”, and “technical type”.

The path of the supportive type is as follows: ~ PM * RM * SM * ~NS * LS * FM. This type indicates that the hospital CFO can build organizational vision, timely adjust the strategic goals of the hospital, master and understand the resources of the organization, rationally allocate resources to support the development of different projects and departments and motivate people to complete the organizational goals. Compared with CFOs in commercial organizations, hospital CFOs are primarily concerned with national health policy rather than commercial interests in formulating organizational strategy. Hence, the CFO should supervise departments to improve healthcare delivery and focus on supporting programs that improve healthcare services.

The path of interpersonal type is as follows: PM * RM * LA * NS * LS * ~FM. It emphasizes the abilities and skills to build good relationship with internal and external people to unite organizational members and coordinate external forces to achieve organizational goals. Hospital CFO plays significant role in corporate leadership, requiring abilities to lead in devising and implementing the hospital’s operational planning processes to ensure that strategic objectives are cascaded down to all levels of organization members [3]. As the participant indicated: “China’s health system is medically dominated, many clinical experts lack the awareness and concept of organizational management, and as a CFO, I need to work to build a relationship of mutual trust with them, to ensure the execution and implementation of organizational strategies.” (*Participant 12*).

The path of all-around development type is as follows: RM * SM * LA * NS * LS. This path refers to five core competencies and highlights the abilities to provide expertise and advice to hospital management team, to have knowledge of health sector and develop relevant strategies and implement resource allocation, and the skills to the develop and maintain good relationships with internal and external stakeholders in healthcare. In China’s hospital, the ‘clinician turned manager’ phenomenon is widespread [44], hence, as an expert in financial management, the CFO needs to provide strategy direction and professional knowledge to help the management team make finance decisions. Additionally, the hospital CFO is responsible for using financial management skills to deliver financial insight to partners, service users and colleagues. This requires abilities and skills to lead and build good relationship with others.

The path of the technical type is as follows: PM * RM * SM * LA * ~NS * FM. This type highlights the skills of planning, monitoring and controlling to accomplish tasks efficiently and reliably. CFOs must maintain and develop their knowledge and skills and stay abreast of emerging technologies and techniques [2]. Hence, the technical type requires the CFO to possess learning ability. Additionally, CFOs in this path have specialized knowledge and abilities in strategic management, resource allocation, financial management to fulfill their role as finance experts, generalists, performance leaders, and growth champions [45–47].

### Theoretical contributions

Research on hospital CFO remains an under-explored topic in the literature. This study fills this gap and provides several theoretical contributions. First, research on healthcare system has highlighted the significant role played by hospital CFOs in improving hospital management and healthcare services [1, 2]; it offers little to reveal how to become an excellent hospital CFO. To our best knowledge, this is the first study to identify effective implementation pathways to becoming an excellent hospital CFO. We found that a single competency is not a necessary condition to being an excellent hospital CFO, but requiring a combination of multiple competencies. In contrast to traditional planar research, we provide a three-dimensional perspective to reveal the mechanisms that affect CFO performance excellence.

Second, we applied the QCA method to competency research in healthcare and broadened the choice of research methods for studies of human resources topics. There is rarely a single factor that determines the outcome. By introducing the QCA method into competency research, we revealed a single competency factor is not a necessary condition to become an excellent hospital CFO. We proposed various combined pathways leading to excellent performance for hospital CFOs. This provides a new method to assess the correlation between competency and hospital CFOs’ performance.

### Implications

This research also provided practical implications for hospitals and their CFOs. First, it is necessary and challenging for hospitals to set up the CFO position and find suitable candidates. Although the National Health Commission of China proposes the qualification required by hospital CFO, this is just the basic requirement, not enough to meet the needs of the position, resulting in many hospital CFOs does not having ability to perform their responsibilities. This research identified seven core competencies required by hospital CFOs and proposed four effective implementation pathways to becoming excellent hospital CFOs, presenting a detailed competency portrait to describe excellent hospital CFOs in China. The proposed competencies and implementation pathways can be used as a guide in selecting, training and evaluating hospital CFOs. For example, the China Association of Chief Financial Officers can cooperate with universities to develop relevant training courses to strengthen the improvement of hospital CFOs in specific competencies. Additionally, hospital may also use the psychological tools to evaluate those competencies of candidates in the recruitment of CFOs.

Second, to improve hospital management and operation, a growing number of public hospital in China have established the position of CFO. However, the cognition of the position of hospital CFO and the exploration of an excellent hospital CFO are rare but necessary. In this study, we proposed four implementation pathways to become excellent hospital CFOs rather than a single perspective. It clearly depicts the portraits of excellent hospital CFO to help people better understand the hospital CFOs in China. Additionally, due to the different internal and external environments faced by organizations, even the same position requires different competencies in different organizations [41]. Hence, hospitals can select the suitable type of CFOs and train them by relevant pathway according to the needs of organizations.

Third, this study indicates that a single competency factor is not a necessary factor to becoming an excellent hospital CFO and proposes four paths as effective implementation paths rather than a single optimal equilibrium. Additionally, while the competencies are important, they are not easily taught but rather acquired through lengthy experience in the field. So far, hospital CFOs in China must be qualified accountants who are members of either the senior accountant or the certified public accountant classification, policy makers should establish a variety of conditions as the basic requirements to assess the various competencies of the CFO, rather than just the requirement of professional certification.

Ultimately, the proposed competencies and effective pathways can also serve as a career blueprint and a guide to self-training and development for potential CFOs. For example, several competencies such as learning ability, leadership skill and resource management ability are code conditions in most implementation pathways, and individuals could develop themselves in those competencies.

### Limitation and future research

Despite the enormous effort we have invested, this study, as the pioneer study to explore the competency of hospital CFOs in a holistic perspective based on the QCA method, still has the several limitations and provides opportunities for further studies in the future. First, the samples for this study were all from the comprehensive hospitals. As organizational contexts are different, future research can verify and expand on the results in specialized hospitals, such as the children’s hospital and oncology hospital. Second, this study only focused on the samples in China; in the future, it is necessary to explore samples from medical developed countries such as the United States, United Kingdom and Australia, to enrich the findings. Third, our study is the first application of the QCA method in the research of hospital CFO to identify core competency and effective implementation pathways to develop excellent CFO; future research can expand this result, combing QCA and the three-level coding of grounded theory to explore a more detailed multi-pathways competency framework for hospital CFO. Fourth, the results of coding may be subjectively affected by coders. However, the coders have been trained before coding, and the necessary results have been verified by experts, which could reduce the influence of subjectivity. Fifth, the scores around the competencies have been assigned by the researchers according to a precise formula and verified by experts, it still limited rely on the interviewees self-assessing their competencies, because they are suspected of potentially glorifying their competencies. In the future, it is necessary to expand the sample size or develop an objective competency assessment system to supplement the findings of this study. Sixth, as QCA is emerging in human resources management and this study primarily explored the effective pathway of excellent hospital CFO in China, it is limited to assessing the intervention effect of proposed pathways. Hence, future studies can design intervention tests based on the competency proposed by this study and explore the intervention effect on the performance of hospital CFOs. Additionally, this study is only the starting point for exploring the competency of excellent hospital CFO; we welcome future research to explore specific programs to improve those competencies for training or hiring practices of hospital CFO by combing psychology, management science and other disciplines to providing the comprehensive research for improving management performance of hospital CFO to deal with challenges faced in China and worldwide.

## Conclusions

The responsibilities of hospital CFOs are complex and varied, ranging from statutory duties relating to accountability to corporate strategic management and operational management. A better understanding of competencies required by CFOs is essential to implement their responsibilities effectively. As a starting point for this process, we identified core competencies required by hospital CFOs and proposed several effective implementation pathways to become excellent hospital CFOs in China. This study pioneered the introduction of the QCA method to explore competency of hospital CFOs and provides both theoretical and practical implications.

### Electronic supplementary material

Below is the link to the electronic supplementary material.

Supplementary Material 1

Supplementary Material 2

## Data Availability

The data and materials analyzed during the current study are not publicly available because they are part of datasets for other further papers, but the interview materials (questions) are available from the corresponding author on reasonable request.
