# Supplementary material for: Exploring effective implementation pathways to become an excellent chief financial officer in public hospital: a qualitative comparative analysis (QCA) from China
Source: BMC Health Serv Res. 2024 Jan 23;24:124. doi: 10.1186/s12913-024-10588-x (PMC10804516; doi:10.1186/s12913-024-10588-x)
Supplement: Supplementary file 1 — Supplementary Material 1 [file 12913_2024_10588_MOESM1_ESM.docx]

**Appendix 1: Semi-structured Interview Guides in Stage 1**

Prior to beginning the interview, each participant was provided with a summary of this study, including study background, research aim and objectives.we also provided a short introduction to the concept of competency. The interviewer then used the following questions to guide the interview.

**Interview questions:**

1. Please describe your understanding of hospital CFOs position.
2. Please describe what are excellent hospital CFOs in your opinions.
3. Please describe the competency necessary for hospital CFOs to perform their job duties and responsibilities and explain those items.
4. Do you have any other points to add on this topic?
