# Supplementary material for: Exploring effective implementation pathways to become an excellent chief financial officer in public hospital: a qualitative comparative analysis (QCA) from China
Source: BMC Health Serv Res. 2024 Jan 23;24:124. doi: 10.1186/s12913-024-10588-x (PMC10804516; doi:10.1186/s12913-024-10588-x)
Supplement: Supplementary file 2 — Supplementary Material 2 [file 12913_2024_10588_MOESM2_ESM.docx]

**Appendix 2: Semi-structured Interview Guides in Stage 2**

Prior to beginning the interview, each participant was provided with a summary of this study, including study background, research aim and objectives. We also provide a list including seven competencies for each interviewer. The interviewer then used the following questions to guide the interview.

**Interview questions:**

1. Please introduce yourself in detail, such as age, education background, working age and work experience.
2. Please describe your understanding of hospital CFOs position and share your work experience in this position.
3. This is the core competencies list for hospital CFOs including personal morality, resource management, strategy management, learning ability, negotiating skill, leadership skill and financial management. Could you please describe your understanding of those competencies?
4. Could you please provide evidence to explain whether you possess those competencies, and describe how you implement those competencies in your work?
5. Do you have any other points to add on this topic?
